# Supplementary material for: Burnout Among Hospital Nurses in Kazakhstan
Source: Nurs Rep. 2025 Mar 6;15(3):92. doi: 10.3390/nursrep15030092 (PMC11946353; doi:10.3390/nursrep15030092)
Supplement: Supplementary file 1 [file nursrep-15-00092-s001.zip › nursrep-3428885-supplementary.pdf]

Table S1. Distribution of development levels of three main burnout indicators (EE, DP, PA) among hospital nurses in Kazakhstan depending on age, work experience and position (*n*=284).

		low			moderate			high		
		Emotional exhaustion, %	Depersonalization, %	Personal accomplishment, %	Emotional exhaustion, %	Depersonalization, %	Personal accomplishment, %	Emotional exhaustion, %	Depersonalization, %	Personal accomplishment, %
Age	21–30 years	7.75	4.93	11.27	6.69	3.52	2.11	9.86	15.85	10.91
	31–40 years	17.6	4.93	11.62	3.87	5.28	4.22	3.87	15.14	9.51
	41–50 years	9.86	7.04	16.9	10.21	5.99	3.87	13.74	20.77	13.03
	51–60 years	9.86	2.82	8.45	4.93	4.58	2.83	1.76	9.15	5.28
Total		45,07	45.07	19.72	48.24	25.7	19.37	13.03	29.23	60.91
Work experience	3–10 years	13.38	5.99	17.61	9.86	6.69	3.52	13.38	23.95	15.49
	11–20 years	16.2	6.34	13.73	6.69	4.58	3.87	5.28	17.25	10.57
	21–30 years	8.45	5.28	11.62	7.04	3.52	4.23	9.87	16.9	8.8
	31-40 years	6.34	2.11	4.22	1.41	3.52	1.41	0.7	2.46	3.52
	>40 years	0.7	0	1.06	0.7	1.06	0	0	0.35	0.35
Total		45,07	45.07	19.72	48.24	25.7	19.37	13.03	29.23	60.91
Work position	Nurse	34.86	14.09	38.73	20.42	13.39	9.16	22.54	50.0	29.93
	Advanced practice nurse	3.17	2.46	2.47	1.76	1.41	0	1.41	2.46	3.87
	Senior nurse	5.99	2.82	6.69	3.52	3.87	3.87	5.28	8.45	4.23
	Head nurse	0.35	0	0	0	0.35	0	0	0	0.35
	Deputy director of nursing service	0.7	0.35	0.35	0	0.35	0	0	0	0.35
Total		45,07	45.07	19.72	48.24	25.7	19.37	13.03	29.23	60.91
